# 2-Nitro-1,3-dinitro­oxypropane

**DOI:** 10.1107/S1600536813004170

**Published:** 2013-02-16

**Authors:** Megan M. Breiner, David E. Chavez, Damon A. Parrish

**Affiliations:** a MS C920, Los Alamos National Laboratory, Los Alamos, NM 87545, USA; b Technical Staff Member, MS C920, Los Alamos National Laboratory, Los Alamos, NM 87545, USA; c CBMSE, Code 6910, Naval Research Laboratory, Washington, DC 20375, USA

## Abstract

The title compound, C_3_H_5_N_3_O_8_, was synthesized by reacting 2-nitro­propane-1,3-diol with acetyl nitrate. The mol­ecule is bisected by a crystallograpic mirror plane. In the crystal, the mol­ecules pack in a ribbon-like fashion along the *c* axis, with the central nitro groups pointing in the same direction. C—H⋯O contacts apparently provide some additional packing stabilization.

## Related literature

Nitrate esters are often studied for their energetic materials properties. For example, we have reported the synthesis and crystal structure of a low melting nitrate ester (Chavez, *et al.* 2008 ▶)·The title compound was first synthesized by Römer (1955) ▶ but no information has been reported on the crystal structure of this material. A smilar structure was reported that differs only in a nitro­oxy group at the 2-position (Espenbetov *et al.* 1984 ▶).
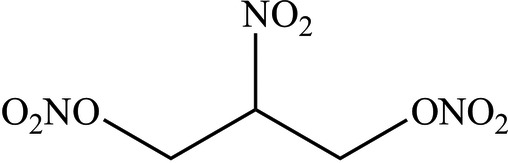


## Experimental

### 

#### Crystal data

C_3_H_5_N_3_O_8_
*M* *_r_* = 211.10
Orthorhombic, 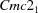

*a* = 14.046 (5) Å
*b* = 9.607 (5) Å
*c* = 5.903 (3) Å
*V* = 796.5 (7) Å^3^
*Z* = 4
Mo *K*α radiation
μ = 0.18 mm^−1^
*T* = 293 K
0.38 × 0.02 × 0.01 mm

#### Data collection

Bruker SMART APEXII CCD diffractometer
Absorption correction: multi-scan (*SADABS*; Bruker, 2008 ▶) *T*_min_ = 0.935, *T*_max_ = 0.998
3416 measured reflections
841 independent reflections
587 reflections with *I* > 2σ(*I*)
*R*_int_ = 0.052

#### Refinement

*R*[*F* ^2^ > 2σ(*F* ^2^)] = 0.038
*wR*(*F* ^2^) = 0.083
*S* = 1.00
841 reflections
70 parameters
1 restraint
H-atom parameters constrained
Δρ_max_ = 0.15 e Å^−3^
Δρ_min_ = −0.18 e Å^−3^

### 

Data collection: *APEX2* (Bruker, 2009 ▶); cell refinement: *SAINT* (Bruker, 2009 ▶); data reduction: *SAINT* and *XPREP* (Bruker, 2008 ▶); program(s) used to solve structure: *SHELXTL* (Sheldrick, 2008 ▶); program(s) used to refine structure: *SHELXTL*; molecular graphics: *SHELXTL*; software used to prepare material for publication: *SHELXTL*.

## Supplementary Material

Crystal structure: contains datablock(s) global, I. DOI: 10.1107/S1600536813004170/ld2094sup1.cif

Structure factors: contains datablock(s) I. DOI: 10.1107/S1600536813004170/ld2094Isup2.hkl

Supplementary material file. DOI: 10.1107/S1600536813004170/ld2094Isup3.cml

Additional supplementary materials:  crystallographic information; 3D view; checkCIF report

## Figures and Tables

**Table 1:** Hydrogen-bond geometry (Å, °)

*D*—H⋯*A*	*D*—H	H⋯*A*	*D*⋯*A*	*D*—H⋯*A*
C5—H5*B*⋯O4^i^	0.97	2.56	3.405 (5)	145

Symmetry code: (i) 
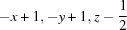
.

**Fig. 1.**
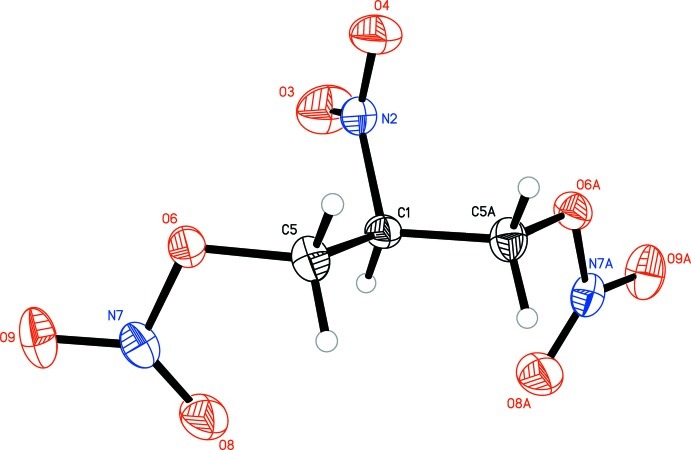
View of 1 showing the labeling of the non-H atoms. Thermal ellipsoids are shown at the 50% probability level.

**Fig. 2.**
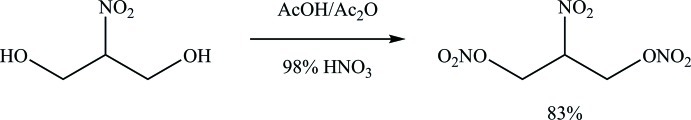
The synthesis of 2-nitro-1,3-dinitrooxypropane.

## supplementary crystallographic information

### Comment

In our efforts to synthesize energetic materials with novel properties, we identified the title compound, 2-nitro-1,3-dinitrooxypropane (Fig. 1), as a nitrate ester for which very little information exists in the literature. The compound was synthesized in a one- step process whereby 2-nitropropane-1,3-diol was subjected to nitration conditions using acetyl nitrate as the substrate (Fig. 2). Suitable crystals were grown from carbon tetrachloride and subjected to X-ray analysis for structure confirmation. The molecule lies on a mirror plane, making only half of the molecule crystallographically unique.

### Experimental

Crystals suitable for X-ray crystallographic analysis were grown from carbon tetrachloride. The crystals were isolated as white needles.

### Refinement

The full-matrix least-squares refinement on F2 included atomic coordinates and anisotropic thermal parameters for all non-H atoms. The H atoms were included using a riding model.

### Crystal data

**Table:**

C_3_H_5_N_3_O_8_	*F*(000) = 432
*M**_r_* = 211.10	*D*_x_ = 1.760 Mg m^−^^3^
Orthorhombic, *C**m**c*2_1_	Mo *K*α radiation, λ = 0.71073 Å
*a* = 14.046 (5) Å	µ = 0.18 mm^−^^1^
*b* = 9.607 (5) Å	*T* = 293 K
*c* = 5.903 (3) Å	Needle, colourless
*V* = 796.5 (7) Å^3^	0.38 × 0.02 × 0.01 mm
*Z* = 4	

### Data collection

**Table:**

Bruker SMART APEXII CCD diffractometer	841 independent reflections
Radiation source: fine focus sealed tube	587 reflections with *I* > 2σ(*I*)
Graphite monochromator	*R*_int_ = 0.052
ω scans	θ_max_ = 26.3°, θ_min_ = 2.6°
Absorption correction: multi-scan (*SADABS*; Bruker, 2008)	*h* = −17→17
*T*_min_ = 0.935, *T*_max_ = 0.998	*k* = −11→11
3416 measured reflections	*l* = −7→7

### Refinement

**Table:**

Refinement on *F*^2^	Primary atom site location: structure-invariant direct methods
Least-squares matrix: full	Secondary atom site location: difference Fourier map
*R*[*F*^2^ > 2σ(*F*^2^)] = 0.038	Hydrogen site location: inferred from neighbouring sites
*wR*(*F*^2^) = 0.083	H-atom parameters constrained
*S* = 1.00	*w* = 1/[σ^2^(*F*_o_^2^) + (0.0389*P*)^2^] where *P* = (*F*_o_^2^ + 2*F*_c_^2^)/3
841 reflections	(Δ/σ)_max_ < 0.001
70 parameters	Δρ_max_ = 0.15 e Å^−^^3^
1 restraint	Δρ_min_ = −0.18 e Å^−^^3^

### Special details

**Table:**

Experimental. Acetic acid (6.25 ml) and acetic anhydride (6.25 ml) were added to a 50 ml jacketed flask. The solution was then cooled to 0 degress C and HNO3 (4.25 g, 98%) was added dropwise while maintaining the reaction temperature below 5 degrees C. The reaction was allowed to stir for 20 min. and 2-nitro-1,3-propanediol (1.51 g, 12.5 mmol) was added. After stirring for 2 h at 0 degrees C, the temperature was raised to 20 degrees C over one hour and then stirred at 20 degrees C for an additional hour The reaction mixture was then poured into 25 ml of ice water and stirred. The white solid was filtered and washed with water and air dried to give 2.19 g of crude 1. This material was then recrystallized from carbon tetrachloride to give white needles. The melting point was measured to be 68–69 degrees C. IR analysis (KBr), proton NMR analysis (300 MHz, deuterioacetone), and elemental analysis were also performed for additional characterization.
Geometry. All e.s.d.'s (except the e.s.d. in the dihedral angle between two l.s. planes) are estimated using the full covariance matrix. The cell e.s.d.'s are taken into account individually in the estimation of e.s.d.'s in distances, angles and torsion angles; correlations between e.s.d.'s in cell parameters are only used when they are defined by crystal symmetry. An approximate (isotropic) treatment of cell e.s.d.'s is used for estimating e.s.d.'s involving l.s. planes.
Refinement. Refinement of *F*^2^ against ALL reflections. The weighted *R*-factor *wR* and goodness of fit *S* are based on *F*^2^, conventional *R*-factors *R* are based on *F*, with *F* set to zero for negative *F*^2^. The threshold expression of *F*^2^ > σ(*F*^2^) is used only for calculating *R*-factors(gt) *etc*. and is not relevant to the choice of reflections for refinement. *R*-factors based on *F*^2^ are statistically about twice as large as those based on *F*, and *R*- factors based on ALL data will be even larger.

### Fractional atomic coordinates and isotropic or equivalent isotropic displacement parameters (Å^2^)

**Table:**

	*x*	*y*	*z*	*U*_iso_*/*U*_eq_	
C1	0.5000	0.2314 (4)	0.4290 (7)	0.0314 (8)	
H1	0.5000	0.1318	0.3932	0.038*	
N2	0.5000	0.2530 (4)	0.6828 (6)	0.0392 (8)	
O3	0.5000	0.1504 (4)	0.8026 (5)	0.0743 (10)	
O4	0.5000	0.3716 (4)	0.7543 (5)	0.0606 (9)	
C5	0.58746 (15)	0.3011 (3)	0.3296 (5)	0.0386 (6)	
H5A	0.5877	0.2874	0.1669	0.046*	
H5B	0.5839	0.4004	0.3581	0.046*	
O6	0.67514 (11)	0.24806 (16)	0.4225 (4)	0.0404 (5)	
N7	0.70956 (17)	0.1273 (2)	0.3130 (4)	0.0429 (6)	
O8	0.65815 (14)	0.07181 (19)	0.1797 (5)	0.0614 (6)	
O9	0.78766 (13)	0.0968 (2)	0.3745 (4)	0.0611 (7)	

### Atomic displacement parameters (Å^2^)

**Table:**

	*U*^11^	*U*^22^	*U*^33^	*U*^12^	*U*^13^	*U*^23^
C1	0.0337 (18)	0.028 (2)	0.032 (2)	0.000	0.000	−0.0047 (17)
N2	0.0324 (16)	0.045 (2)	0.040 (2)	0.000	0.000	−0.004 (2)
O3	0.105 (3)	0.071 (2)	0.047 (2)	0.000	0.000	0.0159 (19)
O4	0.065 (2)	0.057 (2)	0.060 (2)	0.000	0.000	−0.0235 (17)
C5	0.0322 (14)	0.0359 (14)	0.0477 (17)	0.0026 (11)	0.0018 (13)	0.0015 (14)
O6	0.0318 (9)	0.0415 (11)	0.0480 (11)	0.0025 (8)	−0.0019 (9)	−0.0094 (10)
N7	0.0355 (12)	0.0428 (14)	0.0505 (14)	0.0019 (12)	0.0096 (12)	0.0041 (13)
O8	0.0554 (13)	0.0563 (14)	0.0725 (15)	0.0030 (11)	0.0032 (14)	−0.0254 (15)
O9	0.0359 (11)	0.0679 (15)	0.0796 (17)	0.0153 (9)	0.0032 (11)	0.0177 (12)

### Geometric parameters (Å, º)

**Table:**

C1—N2	1.512 (6)	C5—O6	1.441 (3)
C1—C5	1.517 (3)	C5—H5A	0.9700
C1—C5^i^	1.517 (3)	C5—H5B	0.9700
C1—H1	0.9800	O6—N7	1.413 (3)
N2—O3	1.213 (4)	N7—O9	1.192 (3)
N2—O4	1.215 (4)	N7—O8	1.194 (3)
			
N2—C1—C5	108.8 (2)	O6—C5—H5A	109.0
N2—C1—C5^i^	108.8 (2)	C1—C5—H5A	109.0
C5—C1—C5^i^	108.2 (3)	O6—C5—H5B	109.0
N2—C1—H1	110.3	C1—C5—H5B	109.0
C5—C1—H1	110.3	H5A—C5—H5B	107.8
C5^i^—C1—H1	110.3	N7—O6—C5	114.1 (2)
O3—N2—O4	124.0 (4)	O9—N7—O8	130.4 (2)
O3—N2—C1	117.8 (3)	O9—N7—O6	112.1 (2)
O4—N2—C1	118.2 (3)	O8—N7—O6	117.5 (2)
O6—C5—C1	112.9 (2)		
			
C5—C1—N2—O3	−121.2 (2)	C5^i^—C1—C5—O6	176.71 (15)
C5^i^—C1—N2—O3	121.2 (2)	C1—C5—O6—N7	85.4 (3)
C5—C1—N2—O4	58.8 (2)	C5—O6—N7—O9	171.0 (2)
C5^i^—C1—N2—O4	−58.8 (2)	C5—O6—N7—O8	−9.7 (3)
N2—C1—C5—O6	58.6 (3)		

Symmetry code: (i) −*x*+1, *y*, *z*.

### Hydrogen-bond geometry (Å, º)

**Table:**

*D*—H···*A*	*D*—H	H···*A*	*D*···*A*	*D*—H···*A*
C5—H5*B*···O4^ii^	0.97	2.56	3.405 (5)	145

Symmetry code: (ii) −*x*+1, −*y*+1, *z*−1/2.
